# Genetic Evaluations of Stillbirth for Five United States Dairy Breeds: A Data-Resource Feasibility Study

**DOI:** 10.3389/fgene.2022.819678

**Published:** 2022-04-11

**Authors:** Anil Sigdel, Xiao-Lin Wu, Kristen L. Parker Gaddis, H. Duane Norman, José A. Carrillo, Javier Burchard, Francisco Peñagaricano, João Dürr

**Affiliations:** ^1^ Council on Dairy Cattle Breeding, Bowie, MD, United States; ^2^ Department of Animal and Dairy Sciences, University of Wisconsin-Madison, Madison, WI, United States

**Keywords:** dairy cattle, calving ease, maternal effects, predicted transmitting ability, reliability

## Abstract

Genetic selection has been an effective strategy to improve calving traits including stillbirth in dairy cattle. The primary objectives of the present study were to characterize stillbirth data and determine the feasibility of implementing routine genetic evaluations of stillbirth in five non-Holstein dairy breeds, namely Ayrshire, Guernsey, Milking Shorthorn, Brown Swiss, and Jersey. An updated sire-maternal grandsire threshold model was used to estimate genetic parameters and genetic values for stillbirth. Stillbirth data with the birth years of dams from 1995 to 2018 were extracted from the United States national calving ease database maintained by the Council on Dairy Cattle Breeding. The extracted stillbirth records varied drastically among the five dairy breeds. There were approximately 486K stillbirth records for Jersey and more than 80K stillbirth records for Brown Swiss. The direct and maternal heritability estimates of stillbirth were 6.0% (4.5–7.6%) and 4.7% (3.3–6.1%) in Jersey and 6.8% (3.2–10.5%) and 1.1% (0.6–2.9%) in Brown Swiss. The estimated genetic correlations between direct and maternal genetic effects for stillbirth were −0.15 (−0.38 to −0.08) in Jersey and −0.35 (−0.47 to −0.12) in Brown Swiss. The estimated genetic parameters for stillbirth in these two breeds were within close ranges of previous studies. The reliabilities of predicted transmitting abilities in Jersey and Brown Swiss increased substantially, thanks to the substantial increase in available stillbirth data in the past 10 years. The stillbirth records for Ayrshire, Guernsey, and Milking Shorthorn, which ranged approximately between 3K and 12K, are insufficient to implement reliable routine genetic evaluations of stillbirth in these three dairy breeds. Estimated genetic (co)variances and genetic values deviated considerably from the reported ranges of previous studies, and the reliabilities of predicted transmitting abilities were low in these three breeds. In conclusion, routine genetic evaluations of stillbirth are feasible in Brown Swiss and Jersey. However, reliable genetic evaluations of stillbirth in Ayrshire, Guernsey, and Milking Shorthorn require further data collection on stillbirth.

## Introduction

Stillbirth is a severe economic concern to dairy producers (Lombard et al., 2019). In the United States, stillborn calves are those born dead or dying within 48 h of birth (Weller et al., 1988; Berger et al., 1998). The direct economic cost of stillbirth includes loss of replacement calves and increased veterinary and labor costs. The loss of replacement calves in turn limits selection opportunity, thereby resulting in reduced genetic gains. Stillbirth has long-term effects on dams, including the increased risk of health and fertility problems, compromised animal welfare, and early culling (Berry et al., 2007; Bicalho et al., 2007, 2008). A February 2016 USDA report showed that the average stillbirth rate was 5.6% in the United States with the higher percentage of stillbirths reported in small and medium-dairy herds (6.8 and 6.4% respectively) as compared to the large dairy herds (5.1%) (USDA, 2016). The annual loss due to stillbirth was around $125.3 million per year for the United States dairy industry (Meyer et al., 2001).

Genetic selection has been an effective strategy to improve calving traits and reduce disease incidence over the past century, complementary to management, and clinic methods (Barkema et al., 2015). The contribution of genetic factors to the risk of calving stillborn calves in cattle was investigated in several research studies (Cole et al., 2007a; Heringstad et al., 2007; Yao et al., 2014). Non-trivial genetic variation contributed to stillbirth, though the heritability estimates for stillbirth were often low (Hansen et al., 2004; Cole et al., 2007b; Heringstad et al., 2007; Yao et al., 2014). Genetic evaluations of calving ease, a trait highly genetically correlated with stillbirth, began in 1978 (Berger, 1994; Van Tassell et al., 2003; Cole et al., 2005). However, genetic evaluations of stillbirth were published much later in 2006, and implemented for Holstein cattle only (Cole et al., 2007a). The calving ability index, which included economic values of both stillbirth and calving ease, was added into the 2006 revision of lifetime net merit, the flagship selection index used to rank United States dairy cattle. Yao et al. (2014) studied the feasibility of multi-breed genetic evaluations of stillbirth, involving Brown Swiss, Jersey, and Holstein cattle. The addition of stillbirth information to the lifetime net merit selection index can help improve the profitability of Brown Swiss and Jersey cattle in the United States. So far, official genetic evaluations of stillbirth, either multibreed or single-breed, have not been officially implemented for non-Holstein dairy breeds, primarily due to insufficient stillbirth data.

Threshold models were introduced to genetic evaluations of calving traits such as calving ease (Berger, 1994) because linear models violated the normality assumptions, leading to biased estimates of variance components and genetic parameters (Gianola and Foulley, 1983). Both direct and maternal effects were included in the evaluation model out of consideration for the antagonism between direct and maternal genetic effects on dystocia (Phocas and Laloë, 2003). The direct genetic effect refers to the influence of the calf genotype on its ability to be born alive. The maternal genetic effect is the indirect genetic effect in which the genotype of a dam affects the phenotype of the calf through the environment provided by the dam (Olsen et al., 2010). For the genetic evaluation of calving ease or stillbirth, a sire-maternal grandsire (S-MGS) model increased the accuracy of service sire evaluations by partially accounting for differences in the merit of mates, and it allowed for estimating maternal effects (Van Tassell et al., 2003; Cole et al., 2007a).

The present study represented an effort to leverage the United States dairy data repositories available at the Council on Dairy Cattle Breeding (CDCB) toward the official implementation of genetic evaluations in five non-Holstein dairy breeds, namely Ayrshire (AY), Guernsey (GU), Milking Shorthorn (MS), Brown Swiss (BS), and Jersey (JE). There were four primary tasks: 1) determining the extent to which stillbirth data were recorded in these breeds; 2) characterizing stillbirth data in terms of stillbirth rates and their distributions; 3) performing preliminary single-breed genetic evaluations using an updated S-MGS model in these breeds, and 4) determining the feasibility of routine genetic evaluations of stillbirth for these breeds, given the currently available stillbirth data.

## Materials and Methods

### Data Extraction

**FIGURE 1:**
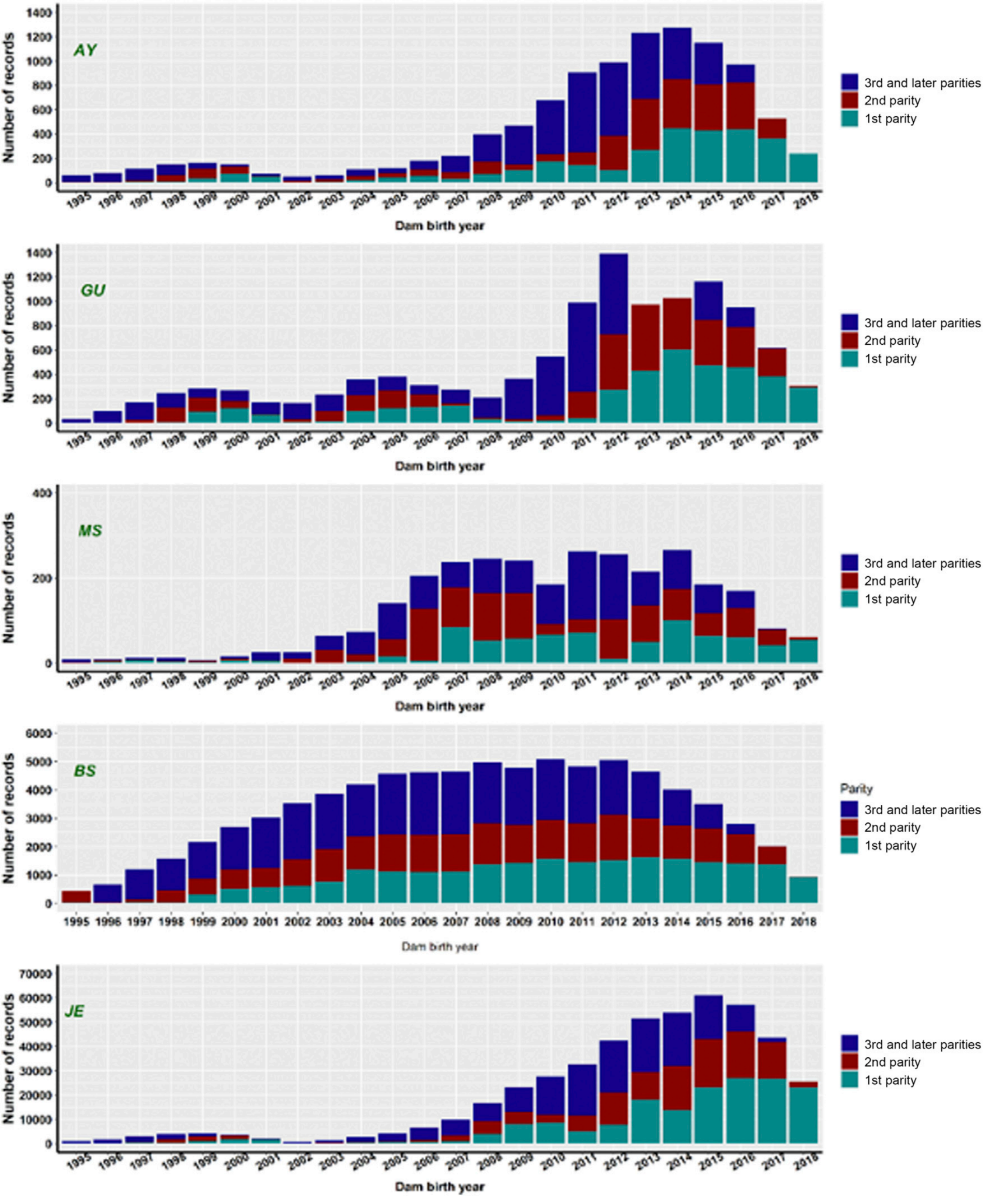
The number of stillbirth records by dam birth year in first, second, third-or-later parities for five non-Holstein dairy breeds. AY = Ayrshire; GU = Guernsey; MS = Milking Shorthorn; BS = Brown Swiss; JE = Jersey.

Records of stillbirth with the birth years of dams ranging from 1995 to 2018 were extracted from the United States national calving ease (CE) database maintained by CDCB (https://www.uscdcb.com/), subject to a series of data quality edits (Van Tassell et al., 2003; Yao et al., 2014). The data included purebred calvings based on breed code of service-sire and dams. Records on single-born calves from five non-Holstein dairy breeds were extracted. In the United States national CE database, stillbirths are reported on a 3-point scale, with scores 1, 2, and 3 representing calves born alive, calves born dead, and calves that died within 48 h of birth, respectively. Since the frequency of score 3 is less, we combined scores 2 and 3 into a single category. Hence, stillbirth was defined as a binary trait in our study with score 1 representing a live-born calf and score 2 representing a calf that was born dead or die within 48-hours of birth. The finalized dataset included only records from those herds that reported at least one case of stillbirth (score 2 or 3) to avoid possible biases from herds that reported only live calves. Contemporary groups such as the herd-year categories ought to have at least five calving records. Dams were allowed to have more than one calving event in the data, but calving events with unknown MGS were eliminated from the analysis. Records from the first, second, third, and later parities with the birth years of dams ranging from 1995 to 2018 were included in the preliminary genetic evaluations. Note that there are fewer records in recent years because dams born in 2018 have not yet reached third or later parities. The number of records with calf livability scores varied widely among different dairy breeds, as reflected by different scales on the *y*-axis (Figure 1). We retained only purebred calving records based on the breed code of dams and service sires.

### Statistical Model

Single-breed genetic evaluations were implemented using a univariate S-MGS threshold model following Van Tassell et al. (2003) and Cole et al. (2007a) but updated with two additional interaction terms that reflects the current national genetic evaluation for stillbirth in United States Holstein cattle. The fixed effects included the year-season of calving, parity-sex of calves, sire birth year, and maternal grandsire birth year, plus two parity-sex-birth-year interaction terms pertaining to sires and MGS, respectively. Including these two interaction terms allowed for capturing a recent trend in calves’ sex ratio, especially in the first lactation animals, because the sex ratio was skewed towards females due to the use of sexed semen in the past decades (Healy et al., 2013). Random effects included herd-year, additive genetic effects of sires, additive genetic effects of maternal grandsires, and random residual effects. The herd-year as a random variable in the model avoids extreme category problems in which all records in a fixed group belong to the same response category (Misztal et al., 1989). Including birth year effects allowed for assessing phenotypic and genotypic trends of stillbirth over time. The updated S-MGS model for genetic evaluation of stillbirth is the following:

$$y_{ijklmnopqr}=hy_{i}+YS_{j}+PS_{k}+sB_{l}+mB_{m}+sPSB_{n}+mPSB_{o}+s_{p}+m_{q}+e_{ijklmnopqr}$$ (1)

Here, $y_{ijklmnop}=$ stillbirth (SB) score for individual *r*, $hy_{i}∼N\left(0,\sigma_{hy}^{2}\right)$ is a random effect of herd-year *i*, $YS_{j}=$ fixed effect of year-season *j*, $PS_{k}=$ fixed effect of parity-sex *k*, $sB_{l}=$ fixed (genetic) effect of sire birth year *l*, $mB_{m}=$ fixed (genetic) effect of MGS birth year *m*, $sPSB_{n}=$ fixed interaction effect of parity-sex-birth year combination *n* pertaining to sires, $mPSB_{o}=$ fixed interaction effect of parity-sex-birth year combination *o* pertaining to MGS, $s_{p}=$ random effect of sire *p*, $m_{q}=$ random effect of MGS *q*, and $e_{ijklmnopqr}=N\left(0,\sigma_{e}^{2}\right)$ is a residual. Herd-years are considered random to avoid the extreme category problems caused when all values for a fixed effect subclass fall in the same category (Harville and Mee, 1984; Misztal et al., 1989). Parities consisted of three categories: first, second, and third parities (records from third and later parities were combined as the third parity). Year-season groups began in October and May. Random sire and MGS effects are assumed to follow a multivariate normal distribution, that is, $MVN\left(0,\;G=\;G_{o}⊗A\right)$ , where $G_{o}$ is 2 × 2 direct-maternal (S-MGS) variance-covariance matrix, ⊗ stands for the Kronecker product of matrices, and A is a pedigree-based additive genetic relationship matrix. In the threshold model, the residual variance is fixed to be 1.

### Estimation of Variance Components and Genetic Parameters

Variance-covariance components and genetic parameters were estimated using all the available data in each breed based on a Bayesian S-MGS threshold model implemented via Markov chain Monte Carlo simulation. The threshold model was computed using THRGIBBS1F90 (Tsuruta and Misztal, 2006). Initial values for sire variances, MGS variances, and the sire-MGS covariances were obtained from national genetic evaluation of stillbirth in Holstein (Cole at al., 2007b). The Markov chain Monte Carlo simulation consisted of 500,000 interactions with the first 100,000 iterations discarded as burn-ins. Posterior samples were thinned every 100th and saved to calculate posterior means and standard deviations of variance components. The convergence of Markov chain Monte Carlo sampling output was visually inspected through trace plots of the location and scale parameters. All the MCMC chains converged quickly and there were no apparent evidence of poor mixing and drastic fluctuations of the posterior samples after the burn-in period. Additional diagnostic tests of convergence were carried out using the R CODA package (http://cran.r-project.org).

Sire variances $\left({\text{σ}}_{\text{s}}^{2}\right)$ , maternal grandsire variances $\left({\text{σ}}_{\text{MGS}}^{2}\right)$ , and the sire-MGS covariance ( $\sigma_{s,mgs}$ ) were estimated from the S-MGS model and then transformed into direct ( $\sigma_{D}^{2\;}$ ) and maternal ( $\sigma_{M}^{2\;}$ ) variances, and the covariance between direct and maternal genetic effect ( $\sigma_{D,M}$ ), as follows:

$$\sigma_{D}^{2\;}=4\sigma_{s}^{2}$$ (2)

$$\sigma_{M}^{2\;}=4\sigma_{mgs}^{2}-4\sigma_{s,mgs}+\sigma_{s}^{2}$$ (3)

$$\sigma_{D,M}=\;4\sigma_{s,mgs}-2\sigma_{s}^{2}$$ (4)

The genetic correlation between direct and maternal genetic effects, denoted by $r_{D,M}$ , was:

$$r_{D,M}=\;\frac{\sigma_{D,M}}{\sqrt{\begin{array}{cc} \sigma_{D}^{2} & \times \;\sigma_{M}^{2} \end{array}}}$$ (5)

The direct ( ${\text{h}}_{\text{D}}^{2}$ ) and maternal ( ${\text{h}}_{\text{M}}^{2}$ ) heritability were then calculated as:

hD2=σD2σP2  (6)

hM2=σM2σP2  (7)

The phenotypic variance on the underlying scale was computed as:

$$\sigma_{P}^{2\;}=\sigma_{s}^{2}+\sigma_{mgs}^{2}+\sigma_{e}^{2}$$ (8)

where the residual variance ( $\sigma_{e}^{2}$ ) was fixed to be 1.

### Predicted Transmitting Ability Estimation

Genetic merit for stillbirth (SB) was reported as PTA for %SB due to direct (maternal) additive effects in heifers in a herd with average management conditions, following Cole et al. (2007a), and computed similarly to that for the percentage of difficult births in heifer presented by Van Tassell et al. (2003). On the underlying scale, the sire birth-year group solution was added to the sire solution, and the MGS birth-year group solution was added to the MGS solution. We then subtract from individual PTA on the underlying scale (i.e., solutions from BLUP and solutions from birth-year groups) the mean PTA of reference base, and the phenotypic base (weighted averages of %SB in heifers by sire/MGS year of birth). The underlying sire and MGS solutions were then converted to the observed scale and named service-sire SB (SSB) and daughter SB (DSB). In this study, the reference base for sire solutions was defined by the group of bulls born between 2011 and 2015, and the reference base for MGS solutions was determined by the group of bulls born between 2006 and 2010. The use of a 5-year average as a base benefited smoothing large year-to-year variation due to limited data and varied number of calves in individual years.

Let *T* be the threshold between SB scores 1 and 2 on the observed scale, and ε be the solution on the underlying scale with fixed sire (or MGS) birth-year solutions added to the sire (or MGS) solution. Then,

Pr(ε>T)=%SB (9)

Next, setting up the base for the animals represented in the appropriate group (indicated by *):

$$\bar{Pr\left(\varepsilon^{*}>T\right)}=\%SB$$ (10)

The above is equivalent to:

$$\%SB^{*}=1-\;\bar{F\left(T-\varepsilon^{*}+c\right)}$$ (11)

where F is the standard normal cumulative density function. Note that a constant *c* is added to achieve the desired base. From (11), we have:

$$T+c=F^{-1}\left(1-\%SB^{*}\right)+\varepsilon^{*}$$ (12)

$F^{-1}$ is the inverse of *F*. Finally, by substituting the above relationship, %SB is computed by:

$$\%SB=1-F\left[-\varepsilon +F^{-1}\left(1-\%SB^{*}\right)+\bar{\varepsilon^{*}}\right]$$ (13)

### Reliability of Stillbirth PTA

Following Van Tassell et al. (2003) and Cole et al. (2005, 2007), we computed reliabilities of stillbirth PTA using only the inverse of diagonal information from the model equation:

$$rel_{i}=1-\frac{d_{i}^{-1}}{\sigma_{a}^{2}}$$ (14)

where $rel_{i}$ is the reliability of sire (MGS) *i*, $d_{i}$ is the diagonal element from the model equation, and $\sigma_{a}^{2}$ is the genetic variance. This above formula ignored sire relationships and the influence of the distribution of sires within fixed and random effects, and it assumed a unity reliability for parents. Still, this approximation is expected to be a reasonable approximation of the true reliabilities because of the low heritabilities and the use of an S-MGS model (Van Tassell et al., 2003).

### Phenotypic and Genetic Trends

Linear phenotypic trends were evaluated by regressing mean %SB in heifers on sire birth years in each of the five dairy breeds. Similarly, linear genetic trends were obtained by regressing mean %SSB and %DSB on the sire or MGS birth year, respectively, in each of the five breeds. Yearly genetic and phenotypic trends were plotted by smoothing splines. The latter are function estimates, which provide a means for smoothing noisy data by balancing a measure of goodness of fit to the noisy data with a derivative-based measure of the smoothness of the estimates (Craven and Wahba, 1979).

## Results and Discussion

### Data Summary and Characterization

The extracted stillbirth records from historical data repositories at CDCB varied drastically among the five dairy breeds (Table 1). There were approximately 486K stillbirth records in the JE cattle, making up 80.0% of the total extracted stillbirth records. The 80,394 stillbirth records for BS cattle, were the second largest, accounting for 13.6% of all extracted stillbirth records. The remaining 4.4% of stillbirth records were shared by AY (∼10,406), GU (∼12,441), and MS (∼3,022). The number of stillbirth records per sex-by-parity group was approximately between 44K and 134K for JE, between 10 and 18K for BS, between 1.5 and 2.2K for AY, between 1.7 and 2.5K for GU, and between 379 and 649 for MS cattle. Compared to Yao et al. (2014), the number of stillbirth records in the present study was approximately 4.6 times as large in JE and 1.5 times as large in BS, respectively.

**TABLE 1:** Distribution of the number of records (N) and the percentage of stillbirth (% SB) by parity-sex combination of calves in five non-Holstein dairy breeds.

	First parity				Second parity				Third-and-later parities			
	Male		Female		Male		Female		Male		Female	
	N	%SB	N	%SB	N	%SB	N	%SB	N	%SB	N	%SB
Ayrshire	1,450	8.62	1,643	8.64	1,328	4.21	1,462	5.60	2,234	6.80	2,289	6.64
Guernsey	1,752	8.84	1,969	9.64	1,751	5.19	1,746	6.24	2,722	4.99	2,501	6.11
Milking shorthorn	379	10.29	398	6.53	499	4.40	523	4.58	649	5.08	574	5.22
Brown swiss	10,003	6.48	13,116	5.31	11,235	4.11	10,957	3.90	17,831	4.62	17,252	4.77
Jersey	44,150	7.57	133,816	5.36	46,351	3.17	82,175	2.85	72,784	3.58	106,328	3.32

Overall, stillbirth rates were highest for primiparous heifers, regardless of calf sex, and lower in later parities. On average, the frequency of stillbirth records ranged between 5.31 and 9.64% in primiparous heifers and between 2.85 and 6.64% in multiparous cows across these five dairy breeds. The higher incidence rate of stillbirth in heifers was due to the increased calving difficulty attributable to their small, immature birth canals (Bures et al., 2008). The reduction in %SB from primiparous to multiparous cows was also observed by previous studies (Johanson and Berger, 2003; Cole et al., 2007a; Yao et al., 2014). Stillbirth rates also differed between male and female calves. In AY, GU, JE, and MS, male calves had higher stillbirth rates (3.17–10.29%) than female calves (2.85–8.64%). Similar results were observed in Holstein cattle (Meyer et al., 2001; Johanson and Berger, 2003; Heins et al., 2006; Cole et al., 2007a; Berry et al., 2007; Dhakal et al., 2013; Mellado et al., 2017). Ghavi Hossein-Zadeh et al. (2008) also reported greater odds of calf stillbirth for male calves as compared to female calves (*p* < 0.001; OR = 2.10) in Iranian Holstein cows. They also reported that for same-sex twin pairs, the odds of calf stillbirth were greater (*p* < 0.01; OR = 1.51; MM vs. FF) for male (21.9%) than for female (16.0%) twin pairs.

Generally speaking, the birth weight of a male calf tends to be heavier than a female calf, which possibly is one of the reasons for more difficult parturition and higher death risk associated with male calves (Johanson and Berger, 2003). Yet, data editing criteria could also have some impacts in the present study. Because each herd needed to have at least 1 case of stillbirth (score 2 or 3) to be included, it was likely that we retained more records with male stillbirths than female stillbirths. However, the GU cattle showed an opposite situation: 4.99–8.84% male %SB versus 6.11–9.64% female %SB. Yao et al. (2014) reported that female calves had a higher %SB than male calves in JE and BS cattle. The discrepancy between our observation and Yao et al. (2014) for JE was possibly attributable to the sampling of stillbirth data. The JE stillbirth data used in the present study was approximately 4.6 times as large as that in Yao et al. (2014). Thus, we regard our results as an update of Yao et al. (2014). Higher %SB in female calves was also reported in an early study of crossbred HO and GU (Touchberry, 1992). Overall, JE had the smallest stillbirth rate (4.21%), and the stillbirth rate in AY was the largest (6.81%). The overall stillbirth rates for the remaining three breeds were 6.70% (GU), 5.75% (MS), and 4.83% (BS), respectively.

**FIGURE 2:**
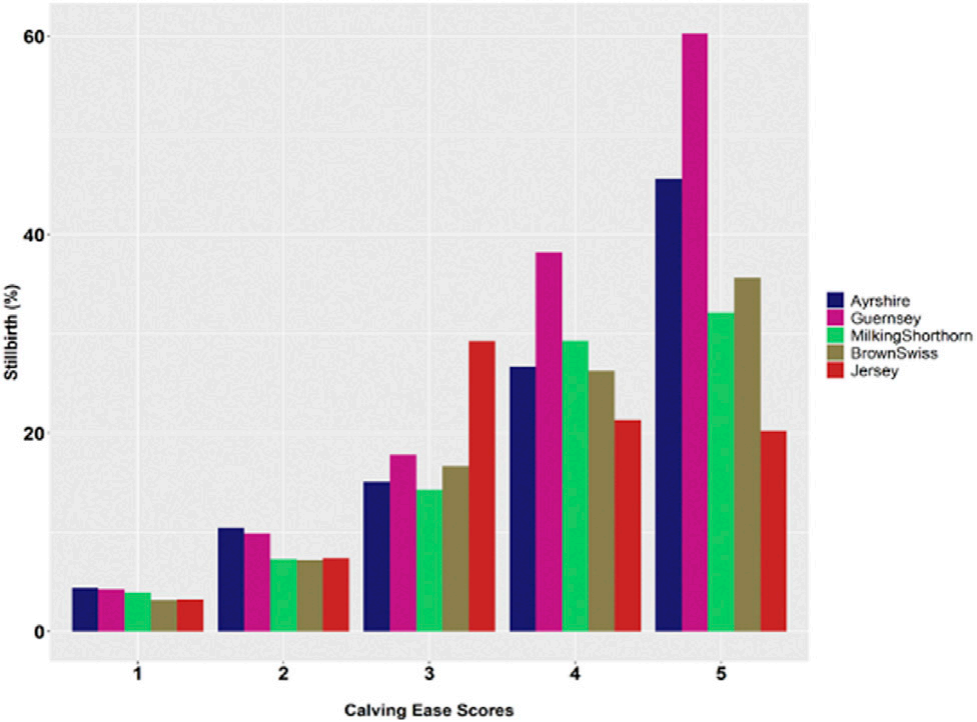
Average population percentage of stillbirth (%SB) by calving ease score in five non-Holstein dairy cattle populations (Calving ease scores: 1 = no problem, 2 = slight problem, 3 = needed assistance, 4 = considerable force, and 5 = extreme difficulty).

Greater calving difficulty (i.e., larger calving ease score) was associated with a higher chance of stillbirths. Figure 2 showed the distributions of stillbirths by calving ease scores in the five non-Holstein dairy breeds. The calving ease scores were the following: 1 = no problem, 2 = slight problem, 3 = needed assistance, 4 = considerable force, and 5 = extreme difficulty. Therefore, the percentage of stillborn calves increased considerably, and consistently as calving difficulty increased, say, approximately from 3 to 4% when calving ease score was 1 (no difficulty) to 30–60% when calving ease score was 5 (extreme difficulty). This observation was consistent with previous studies (Johanson and Berger, 2003; Cole et al., 2007a; Yao et al., 2014). Nevertheless, %SB decreased in JE when the calving ease score was greater than 3 (needed assistance). Similarly, Yao et al. (2014) reported an approximately constant trend (no significant change) from CE = 3 to CE = 5. The JE calves were small compared to Holstein and other dairy calves (Olsen et al., 2010). There is evidence that lower birth weights corresponded to less dystocia and fewer SB (Johanson and Berger, 2003; Berry et al., 2007).

### Estimation of Variance Components and Genetic Parameters

Variance-covariance components of stillbirth were estimated in each of the five non-Holstein breeds before conducting the preliminary genetic evaluations. Table 2 shows the estimated variance-covariance components and genetic parameters in JE and BS. Sire variances are higher than MGS variances in the BS cattle, but both quantities were roughly comparable in the JE cattle. The significantly lower MGS variance in BS could be due to fewer progenies per MGS than that per sire. On average, there were 70 progenies per sire and 20 per MGS, in BS. Hence, there was relatively less information about the maternal genetic effects in the BS dataset. The direct heritability estimates for stillbirth are higher than the maternal estimates in the two dairy populations. In JE cattle, the posterior mean (95% HPD) of direct and maternal heritability of stillbirth were 6.0% (4.5–7.6%) and 4.7% (3.3–6.1%), respectively. For the BS cattle, the estimated direct heritability (95% HPD) for stillbirth was 6.8% (3.2–10.5%), and the estimated maternal heritability (95% HDP) for stillbirth was 1.1% (0.6–2.9%). These heritability estimates were within the previously reported range (1.0–12.0%) for stillbirth heritability (Luo et al., 1999; Meyer et al., 2001; Fuerst and Egger-Danner, 2003; Steinbock et al., 2003; Gevrekçi et al., 2006; Heringstad et al., 2007; Hossein-Zadeh, 2011). In contrast to these findings, Abdelharith (2019), using multi-trait threshold model reported higher heritability estimates ranging between 0.23 and 0.28 for direct genetic effects and 0.35 to 0.39 for maternal genetic effects across first three lactations in Egyptian Friesian cows. However, Yao et al. (2014) reported lower direct and maternal heritability estimates of stillbirth in BS (0.8 and 0.2%, respectively) and JE (0.7 and 1.6%, respectively) cattle. The differences could be due to two facts. Firstly, the stillbirth data used in the present study was significantly larger (i.e., 44–64% larger for BS and 2–5 times larger for JE) than those used in Yao et al. (2014). Our stillbirth data covered the birth years of dams up to 2018, whereas theirs covered the birth years till 2010. Therefore, our data could be more genetically diverse than that used by Yao et al. (2014). Secondly, the updated S-MGS model that we used have two additional interaction terms, which possibly subset a portion of residual variance, leading to higher heritability estimates.

**TABLE 2:** Posterior means (95% HPD) for (co)variance components, heritability, and genetic correlations for brown swiss and jersey, respectively.

Parameters	Brown swiss	Jersey
Sire variance [95% HPD]	0.017 [0.008–0.027]	0.016 [0.012–0.019]
MGS variance [95% HPD]	0.005 [0.001–0.009]	0.014 [0.010–0.017]
Sire-MGS covariance [95% HPD]	0.006 [0.001–0.011]	0.005 [0.002–0.008]
Direct heritability [95% HPD]	0.068 [0.032–0.105]	0.060 [0.045–0.076]
Maternal heritability [95% HPD]	0.011 [0.006–0.029]	0.047 [0.033–0.061]
Direct-maternal genetic correlation [95% HPD]	−0.350 [−0.472 – −0.117]	−0.152 [−0.379 – −0.076]

HPD, highest posterior density interval.

The estimated genetic correlations (%HPD) between direct and maternal genetic effects for stillbirth were −0.35 (−0.47 to −0.12) in BS cattle, and −0.15 (−0.38 to −0.08) in JE cattle. Negative, antagonistic genetic relationships between direct and maternal effects for stillbirth were previously documented. For example, Luo et al. (1999) reported negative genetic correlations of −0.24 between direct and maternal effects of stillbirth in Canadian Holsteins. Steinbock et al. (2003) obtained a genetic correlation of −0.10 between direct and maternal effects of stillbirth in Swedish Holstein. In United States Holstein, the mean genetic correlation between the two effects was −0.02 (Cole et al., 2007b).

The negative genetic correlation between direct and maternal genetic effects for stillbirth reflected a negative relationship between calf size and dam’s pelvic dimension. Selecting on direct effects of stillbirth can lead to small calf size, but it can also result in small heifers that face an increased risk of dystocia and stillbirth calving (Eaglen et al., 2012). Hence, optimal breeding strategies for stillbirth will need to properly weigh the estimated breeding values for both direct and maternal components of stillbirth in the selection index. Still, assortative mating of sires with favorable EBV for maternal stillbirth to heifers can help maintain the pelvic size dimension of heifers (Dekkers, 1994).

Stillbirth heritabilities were over-estimated in the AY, GU, and MS cattle. Direct heritability estimates ranged from 6.8 to 12.9%, and maternal heritability estimates were close to 12% or higher in the AY, GU, and MS cattle. There could be several reasons for these over-estimated heritabilities. A primary reason was due to insufficient stillbirth data in these three dairy breeds. There were between 136 and 395 sires and between 515 and 1,278 MGS for each of these three breeds, but the total number of stillbirth records was between 3,022 and 12,541. Hence, the number of stillbirth records per sire or MGS tended to be very small, and genetic (co) variance components could not be estimated accurately. Secondly, the updated sire-MGS model included two additional interaction terms, which possibly offset the residual variance, thus leading to elevated heritability estimates. The third and probably critical one is that the computed phenotypic variances for the underlying scale, as shown in (8), ignored the interactions, assuming that the mating of a sire to its daughter was rare. The latter, however, was not rare in reality and its impact could be non-trivial, particularly when the data size was small and unbalanced. The estimated genetic correlation between direct and maternal effects of stillbirth was 0.35 in AY, 0.13 in GU, and 0.51 in MS cattle.

### Predicted Transmitting Ability and Reliability of Stillbirth PTA

The mean maternal stillbirth PTA is higher than the mean direct stillbirth PTA. For example, in the JE cattle, the average direct PTA was 5.45%, and the average maternal PTA was 6.40%. Smaller stillbirth PTA suggests a lower stillbirth rate due to favorable direct (maternal) genetic effects, whereas higher maternal PTA for stillbirths indicates a negative or unfavorable genetic contribution. Stillbirth PTA for AY, GU, and MS were also computed, though these (co) variance estimates were not obtained precisely. From a Bayesian perspective, the estimated (co) variance components are treated as priors. Varied prior variances lead to varying shrinkage of the estimated genetic effects, but their orders are mostly retained, given the same dataset. Alternatively, the estimated (co) variance components obtained previously in the same or different breeds (e.g., Cole et al., 2007b) can be used (Cole et al., 2007a, Cole et al., 2007b; Yao et al., 2014). The mean maternal stillbirth PTA (6.01% for AY, 6.55% for GU, and 5.90% for MS) is higher than mean direct stillbirth PTA (4.90% for AY, 5.65% for GU, and 5.25% for MS) in these three breeds.

**FIGURE 3:**
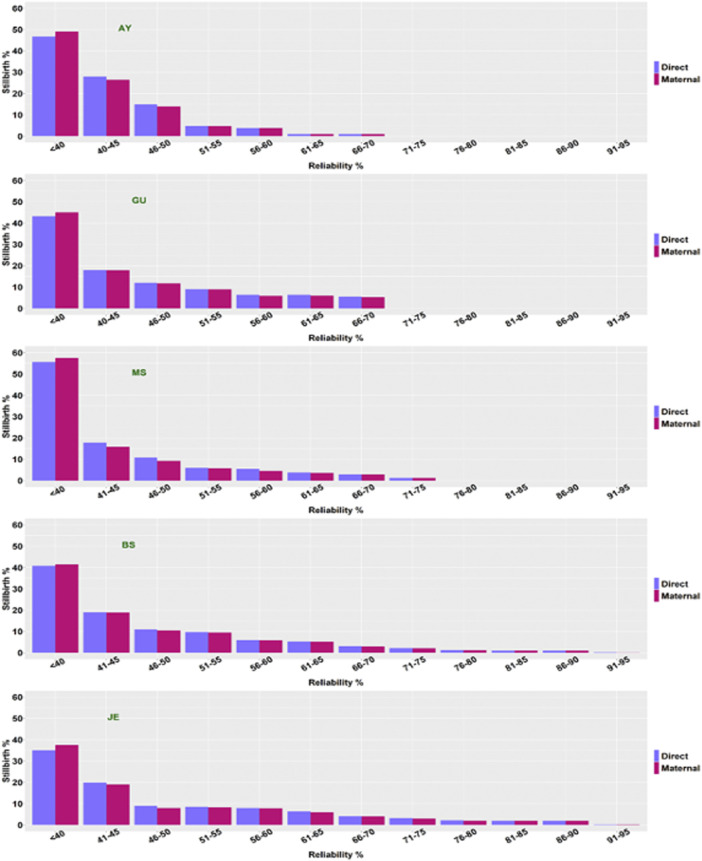
The distributions of reliabilities (%) for direct and maternal PTA for the percentage of stillbirths (%SB) in five non-Holstein dairy breeds. AY = Ayrshire; GU = Guernsey; MS = Milking Shorthorn; BS = Brown Swiss; JE = Jersey.

Reliabilities of stillbirth PTA were estimated for each of the five breeds (Figure 3). The average reliability of direct PTA for stillbirths was higher than that of maternal PTA. A possible reason was that a service sire (sire of calves) tended to have a larger number of offspring than an MGS (sire of dams). The direct (maternal) PTA distribution was heavily right-skewed because most bulls had a smaller number of stillbirth records and very few bulls had many stillbirth records. Consequently, most bulls tended to have low reliabilities. The average reliabilities of direct (maternal) PTA of stillbirth were 48 (47) and 49 (49) in BS and JE, respectively. The average reliabilities of stillbirth for JE in the present study was higher than those for JE reported by Yao et al. (2014) because we included far more data of JE cattle and possibly more progenies per sire (MGS) for the genetic evaluation. Besides that, Yao et al. (2014) reported that more than 60% of JE bulls had PTA with reliabilities less than 40%, but this percentage dropped to below 40% with our results. In other words, the number of JE bulls with limited progeny information has dropped considerably in the present study compared to those in Yao et al. (2014).

The reliabilities of PTA for AY, GU, and MS were lower than those for JE and BS due to insufficient data size. Bulls with PTA of low reliabilities had very limited progeny information. The average reliability of direct (maternal) PTA was 44 (43) for AY, 47 (46) for GU, and 43 (41) for MS, respectively. The percentage of bulls with PTA less than 40% was approximately 48–49% for AY, 43–45% for GU, and 57–59% for MS, respectively. In contrast, approximately 40–41% of the BS sires and 36–37% of the JE sires had less than 40% reliabilities. Possibly, there were sires with limited progeny information. These results call for further stillbirth data collection in AY, GU, and MS before reliable routine genetic evaluations of stillbirth can be implemented in these three breeds. Previous results (Berger, 1994; Cole et al., 2005) have shown that genetic evaluations using a sire model, or an S-MGS model required a large number of effective progenies to achieve high reliabilities. Hence, with continued efforts devoted to more precise and detailed recordings on stillbirth in AY, GU, and MS, we expect a further increase in the reliabilities of stillbirth PTA in these three breeds.

In reality, the computed reliabilities can be inflated for two reasons. Firstly, calves with observations get credit for the underlying scale hertiability instead of actual heritability. This situation holds universally, yet its impact can be minimal because the heritability of stillbirth is low. Secondly, bulls get credit for sire, MGS, and any sons as if their BV were known instead of estimated. This phenomenon was apparent with Holsteins (Van Tassell et al., 2003) but not so with AY, GU, and MS due to sufficient records of stillbirth. Berger (1994) showed sire model evaluation of calving ease requires a large number of effective progenies to achieve high reliabilities. With the simplification in [14] to compute reliabilities, a larger number of effective progenies is probably required under the S-MGS model compared to a sire model (Cole et al., 2005). Distributions of reliabilities of stillbirth PTA in AY, GU, and MS were heavily right-skewed, which reflected lower progeny numbers than were desirable from the perspective of genetic evaluation. Exactly, a few bulls had a large number of records available (and high reliabilities) but most bulls had a very small number of daughters (and low reliabilities). An increase in records will lead to higher reliabilities in these three breeds.

### Phenotypic and Genetic Trends

**FIGURE 4:**
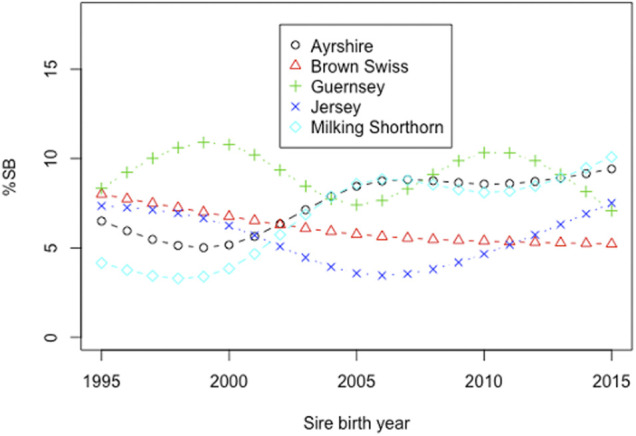
Phenotypic trends for the stillbirth percentage (%SB) in heifers by birth year of sires in five non-Holstein dairy cattle.

Smoothing splines plots of %SB in heifers by the birth year of sires in the five non-Holstein dairy breeds are shown in Figure 4. Linear phenotypic trends were obtained by regressing %SB in heifers on the birth year of sires in each of the five breeds. Negative phenotypic trends were observed in JE and BS. A negative trend in stillbirth is favorable because it represents a reduction of stillbirths over the observation years. Linear regression analysis showed that the phenotypic trend for BS was −0.155 per year and statistically significant from zero (*p* = 0.047). The reduced incidence of stillbirth in BS over the time period was due to correlated response to selection for updated calving ease in BS, thanks to the national genetic evaluation of United States BS (Cole et al., 2007b). Cole et al. (2007b) estimated genetic correlations of 0.67 between direct CE and SB and 0.63 between maternal CE and SB. The decreased incidence of stillbirth could also result from assortative mating of bulls with low PTA for CE to heifers (Mujibi and Crews, 2009). The linear annual phenotypic trend in the JE cattle was −0.013/year, which was not statistically significant from zero (*p* = 0.873). There was no selection for either direct or maternal PTA for stillbirth or a correlated trait such as calving ease in JE cattle. The annual phenotypic trends of stillbirth were all positive and non-significant (*p* > 0.05) in AY, GU, and MS (Figure 4). High year-to-year variations of phenotypic trends were observed for these three breeds, possibly due to insufficient stillbirth records.

**FIGURE 5:**
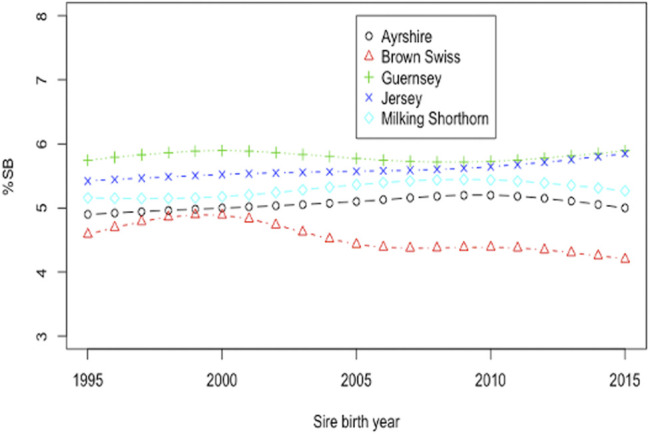
Smoothing spline plot of mean service-sire PTA for the percentage of stillbirths (%SB) by sire birth year since 1995 in five non-Holstein dairy breeds.

**FIGURE 6:**
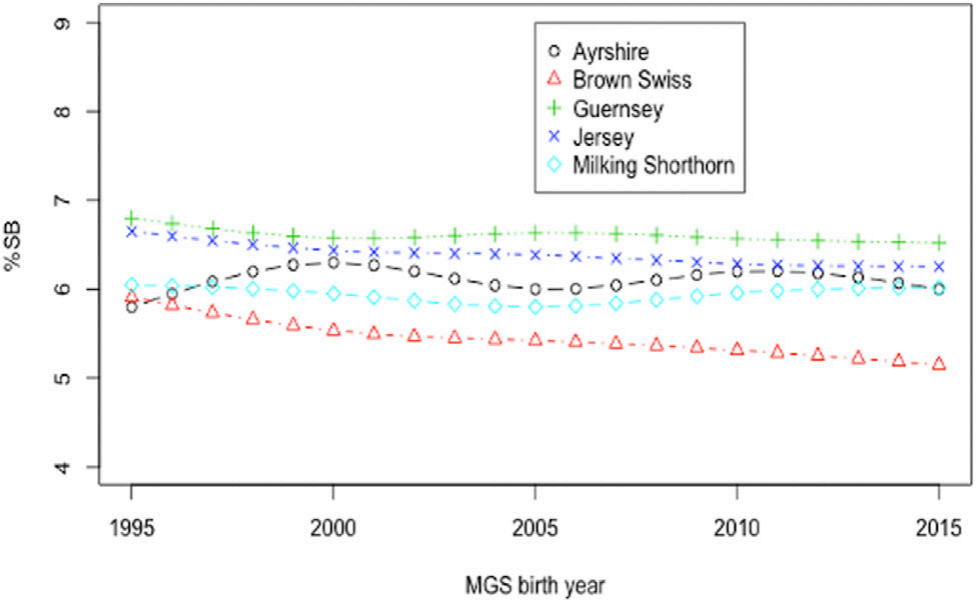
Smoothing spline plots of mean daughter PTA for the percentage of stillbirths (%SB) by maternal grand-sire (MGS) birth year since 1995 in five non-Holstein dairy breeds.

Smoothing splines plots of genetic trends for %SB due to direct and maternal effects are shown in Figure 5 and Figure 6, respectively. Linear genetic trends were obtained by regressing %SSB (or %DSB) on the sire or maternal grandsire (MGS) birth year from 1990 to 2015. In BS cattle, linear annual genetic trend of %SSB was negative (−0.012/year) and significant (*p* = 0.006). The annual genetic trend of %DSB was also negative (-0.027) and significant (*p* = 0.004). A significant, negative genetic trend of stillbirth was favorable because it suggested a significant yearly drop in the stillbirth rate due to favorable genetic effects. The favorable genetic trends in the BS cattle resulted from correlated responses to selection for calving ease, implemented by the United States national genetic evaluation for the BS cattle since 2006 (Cole et al., 2005). Favorable genetic trends for stillbirth in the BS cattle were also reported by Yao et al. (2014). There was unfavorable yet not significant yearly increase in the incidence of stillborn calves in JE cattle due to direct additive genetic effects of the sires of calves (0.0041; *p*-value = 0.5477). The maternal genetic trend of stillbirth in JE was also negative and non-significant (−0.009; *p* = 0.959). These slight favorable genetic trends of stillbirth in JE cattle could result from genetic drifting over generations because there has been no selection officially implemented on stillborn or calving ease in the United States JE cattle. Linear direct and maternal genetic trends were all positive (0.012–0.032/year for SSB and 0.010 to 0.046/year for DSB) and non significant (*p* > 0.05) in AY, GU, and MS. Hossein-Zadeh (2011) estimated genetic trends by regressing yearly mean estimates of breeding values on calving year. The study reported increasing and non-significant genetic trends for stillbirth across first three lactations in Iranian Holstein cows using linear sire model but significant and decreasing trends using threshold sire model indicating that the choice of the model has an effect on capturing the patterns of genetic trends of stillbirth. A significant, positive genetic trend of stillbirth is unfavorable because it represents a significant yearly increase of additive direct genetic effect that often contributes to elevated stillbirth instances. These positive genetic trends were not significant (*p* > 0.05) and could result from insufficient stillbirth data because year-to-year variations of SSB (DSB) were large for these breeds. These results otherwise suggested a need for a more accurate and complete recording of stillbirth data.

## Conclusion

Stillbirth data with dam birth years ranging from 1995 to 2018 were extracted from the United States national CE database maintained by CDCB. The amount of stillbirth data varied drastically among the five non-Holstein dairy breeds. The majority (80.0%) of the extracted stillbirth records were represented by JE, followed by BS (13.6%). The AY, GU, and MS shared the remaining stillbirth records (4.4%). Based on an updated S-MGS mixed-effects model, the estimated genetic parameters (heritability and genetic correlation) for BS and JE were within close ranges with those from previous studies, asserting that stillbirth has a genetic component upon which selection can operate to achieve the expected genetic improvement. The genetic correlations between direct and maternal genetic effects for stillbirth were negative for BS and JE. Hence, both direct and maternal components of stillbirth need to be considered for genetic improvement, e.g., by including PTA due to direct and maternal effects in the selection index for sires. The average reliabilities of direct (maternal) PTA of stillbirth in BS and JE were higher than those reported by Yao et al. (2014), thanks to the substantially accumulated stillbirth data for these two breeds in the recent 10 years. We thus conclude that implementing routine genetic evaluations of stillbirth in these two breeds is feasible and necessary.

Unlike the cases with JE and BS, the stillbirth data sizes for AY, GU, and MS were insufficient for conducting reliable routine genetic evaluations using the S-MGS threshold models. The evidence of data inadequacy was seen in three main aspects. Firstly, genetic parameters in these three dairy breeds were considerably over-estimated and not consistent with previous studies. Secondly, around 43–59% of the evaluated sires had PTA with less than 40% reliabilities in AY, GU, and MS. In contrast, approximately 40–41% of the BS sire and 36–37% of the JE sires had PTA with less than 40% reliabilities. Hence, the sires with limited progeny information were significantly higher in AY, GU, and MS than JE and BS. Thirdly, we observed larger year-to-year variations in the phenotypic and genetic trends in AY, GU, and MS than AY and BS. Thus, our results call for further stillbirth data collection in AY, GU, and MS. Complete and accurate reporting of stillbirth is prerequisite to obtaining unbiased estimates of variance components and genetic parameters, increasing the reliability of PTA for stillbirth, and improving the accuracy of genetic evaluation of stillbirth.

Finally, the present study represented a univariate analysis of stillbirth, following the United States national genetic evaluation of stillbirth in Holstein cattle. Yet, it is worth mentioning that bivariate analyses between stillbirth with a highly genetically correlated trait such as calving difficulty would be beneficial to account for the genetic correlation between the traits and help make the animal breeding strategists (and objectives) more effective (and accurate) with the consideration of a set of economic traits in the breeds under study. It is also worth mentioning that the study is not inclusive but a preliminary one, aiming at leveraging the current data repositories toward official implementing of genetic evaluation of stillbirth in non-Holstein dairy breeds. Hence, in the first place, we would like to know if there are sufficient data to conduct single-trait genetic evaluations of stillbirth in non-Holstein breeds, following the “norm” of the national genetic evaluation of calving ease and stillbirth in Holstein cattle. Still, multiple-breed evaluations of stillbirth will be considered in follow-up studies, which is likely to increase the reliabilities of SSB and DSB to some extent in these breeds.

## Data Availability

The datasets presented in this article are not readily available because The US national calving ease (CE) databases maintained by CDCB are not publicly available. Still, they can be requested to JD, subject to signing an official agreement for non-commercial use only. Requests to access the datasets should be directed to JD, joao.durr@uscdcb.com.
